# Fall Risk Factors and Other Geriatric Syndromes in Older Adults With Diabetes: Experience of a Multidisciplinary Fall Consultation

**DOI:** 10.1155/jdr/6145818

**Published:** 2025-08-21

**Authors:** Aurélie Mailliez, Yaohua Chen, Rachel Litke, Stéphanie Gloria, Hacène Chekroud, Dominique Huvent-Grelle, Liubinka Mirakovska, Cédric Gaxatte, François Puisieux

**Affiliations:** ^1^Department of Geriatrics, CHU Lille, Lille, France; ^2^Univ. Lille, Inserm, CHU Lille, Institut Pasteur de Lille, U1167-RID-AGE-Facteurs de Risque et Déterminants Moléculaires des Maladies Liées au Vieillissement, Lille, France; ^3^Inserm, CHU Lille, Lille Neurosciences & Cognition, UMR-S1172, Degenerative and Vascular Cognitive Disorders, 27023 Univ. Lille, Lille, France; ^4^Nash Family Department of Neuroscience, Friedman Brain Institute, Icahn School of Medicine at Mount Sinai, New York, New York, USA; ^5^Univ Lille, CHU Lille, ULR 2694-METRICS: Assessment of Health Technologies and Medical Practices, Lille, France

**Keywords:** diabetes, falls, geriatric syndromes, older patients

## Abstract

**Aims:** The aim of this study is to assess the prevalence of diabetes in patients attending a multidisciplinary consultation for fall risk assessment and to compare fall risk factors and the prevalence of other geriatric syndromes in patients with and without diabetes.

**Materials and Methods:** A single-center retrospective cohort study was conducted at the Lille University Hospital Geriatrics Department, France. Inclusion criteria were any patients aged 65 years and over consulting for fall risk assessment between January 2, 2005, and January 2, 2015. A comprehensive multidisciplinary clinical evaluation was carried out to establish a personalized assessment of the patient's risk of falling.

**Results:**One thousand five hundred and twenty patients were included. Mean age was 81.4 ± 6.4 years; 72.2% were female, and 20% had diabetes. While patients with diabetes were younger than patients without diabetes (mean age: 79.4 ± 6.1 years vs. 81.9 ± 6.4 years, *p* < 0.001), they were more likely to have had at least two falls in the previous 6 months (65.5% vs. 56.2%; *p* = 0.004), had more balance and gait disorders (respectively, 77.4% vs. 69.7%, *p* = 0.009, and 88% vs. 82%, *p* = 0.012), and had more cognitive decline, urinary disorders, functional dependency, and polypharmacy than patients without diabetes (*p* < 0.0001 for all).

**Conclusions:** Patients with diabetes have more geriatric syndromes and comorbidities, leading to a higher risk of adverse events compared to patients without diabetes even if they are younger. Preventing falls and other geriatric syndromes should therefore be a concern for all healthcare professionals who care for people with diabetes.

**Summary**

• Twenty percent of older adults coming for a fall risk assessment are diabetics.
• Although patients with diabetes are younger than patients without diabetes, they have more geriatric syndromes and comorbidities.
• This exposes them to a higher risk of adverse events, particularly falls at a younger age.

## 1. Introduction

Diabetes mellitus is a major public health issue with a significantly increased prevalence worldwide in recent decades. According to the International Diabetes Federation, 151 million people lived with diabetes in 2000. It reached 415 million in 2015 and is expected to impact 693 million people in 2045, that is, a 4.5-fold increase in 45 years [1]. Type 2 diabetes makes up 90% of the cases [2]. A recent French study showed that the mean age of patients with Type 2 diabetes is 67.6 ± 12.3 years, and more than half are 60–79 years old [3]. Concurrently, the world's population is aging. In 2019, 1 billion people were 60 years or over in the world. According to the World Health Organization (WHO), this number is expected to double, reaching 2.1 billion by 2050. More specifically, the oldest old (i.e., people aged 80 years and over) will even triple by 2050. Therefore, diabetes mellitus is already a common chronic condition in older patients managed by geriatricians, and older patients will increasingly become common patients for diabetologists.

Older patients with diabetes are often more likely to have several comorbid conditions like heart disease, stroke, and disability due to microangiopathic and macroangiopathic complications of diabetes mellitus [4]. Other common chronic conditions called geriatric syndromes can also affect older patients. Their prevalence increases with age, including falls, delirium, cognitive impairment, urinary incontinence, and visual and hearing impairments. All those potential adverse health events could lead to a loss of independence, gait disorders, and decreased quality of life for older patients with diabetes [5–7]. It has been previously shown that patients with diabetes have a higher risk of falls and fractures compared to patients without diabetes [8–10]. Some studies have also shown that diabetes mellitus is associated with other geriatric syndromes, including frailty, malnutrition, sarcopenia, depression, cognitive decline, dementia, or urinary incontinence, which are all potential risk factors for falls [11–13]. But the confirmation of the relationship between diabetes mellitus and geriatric syndromes that increase the risk of falls in older patients who have already fallen remains to be explored. Few real-life data are available on the prevalence of diabetes and geriatric syndromes and their co-occurrence in older patients who are at high risk of falling.

We aimed to examine the frequency of diabetes in patients attending a multidisciplinary consultation for fall risk assessment and to compare the prevalence of fall risk factors, geriatric syndromes, and the recurrence of falls 6 months later in patients with and without diabetes.

## 2. Materials and Methods

### 2.1. Study Design and Participants

A single-center retrospective cohort study was conducted at the Lille University Hospital Geriatrics Department, France. Inclusion criteria were any patients aged 65 years and over consulting for multidisciplinary fall risk assessment between January 2, 2005, and January 2, 2015. Exclusion criteria were refusal to participate. Each patient was successively examined by a geriatrician, a neurologist, a physical medicine and rehabilitation (PM&R) physician, an occupational therapist, and a nutritionist. Patients could also see an ophthalmologist, a social worker, a physiotherapist, a pedicure, and a psychologist, if necessary. This allows a thorough clinical, functional, and environmental evaluation of the patient in order to establish a personalized assessment of a patient's risk of falling.

### 2.2. Data Collection

Data included

• Clinical and sociodemographic characteristics (age; sex; body mass index [BMI]; dwelling place [private home, nursing homes, or long-term care]; type of housing if private home, living alone, or not; educational level; functional dependency; presence of at least one environmental fall risk factor; alcohol; and tobacco use),
• Medical history (diabetes, hypertension, orthostatic hypotension, heart disease, stroke, arthritis, osteoporosis, fractures, chronic respiratory disease, arrhythmia, urinary disorders [including incontinence and urologic diseases], cognitive decline, Parkinson's disease, depression, hearing and visual impairments, peripheral neuropathy, and foot disorders),
• Current medical treatments (including the presence of at least one psychotropic treatment),
• Gait assessment (ability to go out alone, use of a walking aid, proprioceptive, balance or gait disorders, timed up and go (TUG) test [14] completed in over 20 s, and single-leg stance (SLS) [15] test in over 5 s),
• Fall history (number of falls within previous 6 months, presence of fear of falling, and time spent on the ground after a fall).

Polypharmacy and high polypharmacy were defined, respectively, as five and nine or more medications daily. Cognitive functions were assessed with the Mini-Mental State Examination (MMSE). We used an adapted version of MMSE in French from the GRECO (Groupe de Réflexion sur les Evaluations Cognitives) which is a French working group on cognitive assessments. This version is freely available. Cognitive decline was defined when patients had an MMSE score below 24 points out of 30 or had been diagnosed with dementia before the consultation or within 6 months after the consultation, regardless of their MMSE score. The diagnosis of diabetes was made when patients had been previously diagnosed with diabetes or were taking antidiabetic drugs. Hypertension was defined when patients have been diagnosed with hypertension or were taking antihypertensive drugs. Functional dependency was assessed with the activities of daily living (ADL) scale [16]. Environmental fall risk factors were identified from a list, including obstacles on the floor or in the room, poor or slippery floor, insufficient lighting, and unsuitable footwear or clothes.

Physical performance was assessed by observing the patient's gait, performing TUG (test considered abnormal if achieved in more than 20 s) and SLS (test considered abnormal if patient able to hold less than 5 s) tests. Balance disorders were defined as patients losing balance when standing with eyes open or close or patients having difficulties to adapt posture, especially when they underwent a test called “sternal push test” during which the palm of the examiner's hand is lightly pressed against the patient's sternum in a standing position. The fear of falling was assessed by two questions asked to the patient (“Are you afraid of falling?” and “Do you avoid going out for fear of falling?”). If the patient answered “yes” to any of these questions, he or she was defined as having a fear of falling.

Clinical neurological examination by a neurologist included assessment of motor function, sensory function, and deep tendon reflexes to identify peripheral neuropathy. After the multidisciplinary fall consultation, the personal and environmental risk factors and practical therapeutic proposals for managing falls were explained to the patients and their family carers and sent to the patients' general practitioner. Where necessary, physiotherapy sessions tailored to the patient's fall risk factors were prescribed. Patients were assessed 6 months later during a consultation with a geriatrician to inquire about a possible recurrence of falls.

### 2.3. Data Analysis

For quantitative variables, either the Student test or the Pearson correlation test was used. For qualitative variables, variance analysis according to a Fisher test was conducted. The association between two variables was considered significant if the *p* value was less than or equal to 0.05.

Statistical analyses were carried out by the Biostatistics Unit of the Center for Research and Studies in Medical Informatics (CERIM) of Lille University Hospital.

## 3. Results

### 3.1. Description of the Population

**Figure 1:**
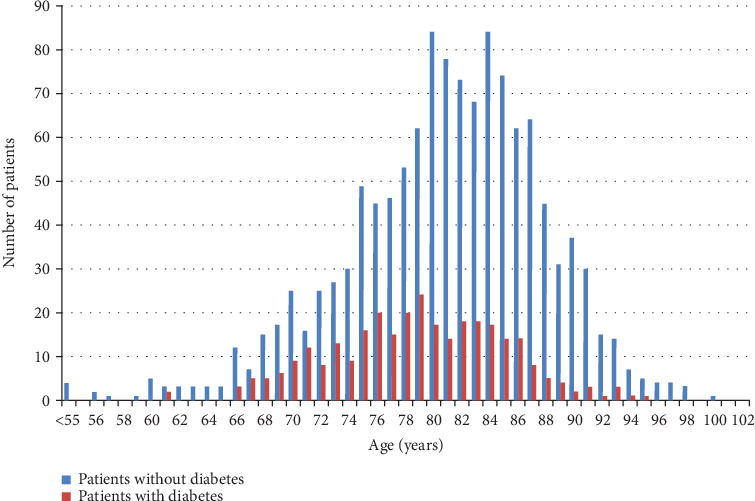
Prevalence of diabetes according to age in the population study.

One thousand five hundred and twenty patients were included in our study. The mean age was 81.4 ± 6.4 years; 72.2% were female. More than half of patients lived alone (52.4%), and 13.2% lived in an institution (Table 1). Three hundred and four patients (20%) had diabetes. As shown in Figure 1, the prevalence of diabetes increased with age to reach a peak at 79 years old and decreased hereafter. However, patients with diabetes were on average younger than patients without diabetes (mean age, respectively, 79.4 ± 6.1 years vs. 81.9 ± 6.4 years, *p* < 0.001). There was no sex difference between patients with diabetes and patients without diabetes. Mean BMI was 26.4 ± 5.2 kg/m^2^. Patients with diabetes had a higher BMI than patients without diabetes (mean BMI, respectively, 28.7 ± 5.5 kg/m^2^ vs. 25.8 ± 4.9 kg/m^2^; *p* < 0.0001). Patients without diabetes were more likely to have a higher level of education than patients with diabetes (43.6% vs. 28.7%; *p* < 0.0001) (Table 1).

### 3.2. Prevalence and Description of Geriatric Syndromes in Patients With Diabetes Versus Patients Without Diabetes

In our study, patients with diabetes had more geriatric syndromes than patients without diabetes (Table 2). Regarding cognitive assessment, patients with diabetes had a lower mean MMSE score of 23.8 ± 5 versus 25 ± 4.8 (*p* < 0.0001) and were more often diagnosed with dementia than patients without diabetes (45.5% vs. 33.4%; *p* < 0.0001). Compared to patients without diabetes, patients with diabetes had urinary disorders more frequently (32.7% vs. 26.2%; *p* = 0.023) and also experienced more functional dependency (mean ADL: 4.8 ± 1.3 vs. 5.2 ± 1.1; *p* < 0.0001). Patients with diabetes were less likely to go out alone (40.5% vs. 49.7%; *p* = 0.004) and used more walking aids (51.5% vs. 45.1%; *p* = 0.047) than patients without diabetes. Patients with diabetes also had lower performances in the SLS test and gait disorders than patients without diabetes (respectively, *p* = 0.017 and *p* = 0.012). Regarding medication, patients with diabetes had polypharmacy (≥ 5 medications) and high polypharmacy (≥ 9 medications) more often (both *p* < 0.0001).

### 3.3. Prevalence and Description of Falls in Patients With Diabetes Versus Patients Without Diabetes

Patients with diabetes were more at risk of having experienced at least two falls in the previous 6 months of the consultation (respectively, 65.5% vs. 56.2%; *p* = 0.004) and had more frequently balance disorder (77.4% vs. 69.7%; *p* = 0.009) compared to patients without diabetes (Table 2).

Eight hundred seventy-four (57.7%) patients returned for the follow-up visit. Among patients with diabetes, 86 (45.5%) experienced new falls between the two consultations versus 346 (43.6%) in patients without diabetes. We did not find any significant difference in terms of recurrence of falls between patients with and without diabetes after a follow-up of 6 months (*p* = 0.63).

## 4. Discussion

In this study, 20% of patients attending the multidisciplinary fall consultation for fall risk assessment had diabetes. Although patients with diabetes were younger than patients without diabetes in terms of chronological age, they experienced more geriatric syndromes and more comorbid conditions, which makes them older in terms of biological age and more at risk of adverse events including falls.

The prevalence of diabetes observed in our population of patients with falls is close to that observed in the general population of comparable age, whether in France, Europe, or the United States [1]. However, given that diabetes is a risk factor for falls, we could have expected an even higher prevalence than in the general population.

We have shown that geriatric syndromes are highly prevalent in older patients with diabetes who have already fallen. Both complications of diabetes like neuropathy or retinopathy and potential hypoglycemia, as well as several geriatric syndromes such as visual and hearing impairments, impaired balance, or depression, are conditions impacting the risk for falls [9, 17, 18]. In our study, we also noted more cognitive decline, gait disorders, urinary incontinence, and polypharmacy in patients with diabetes. Other studies have also demonstrated the greater prevalence of geriatric syndromes in patients with diabetes which are independent risk factors for falls. Rodríguez-Sánchez et al. found a significant association after adjustment between diabetes and both cognitive impairment and depression [19]. Gregg et al. showed a greater incidence of disability in women with diabetes compared to patients without [20]. Cheng et al. suggested that patients with diabetes had a higher risk of mild cognitive impairment, any dementia, vascular dementia, and Alzheimer disease compared to patients without diabetes [21, 22]. A significant correlation has already been demonstrated between cognitive and physical impairments in frail patients suffering from diabetes and hypertension [22]. Diabetes has also been shown to be associated with an increased risk of visual impairment which has an important impact on the risk of falling [23, 24]. Polypharmacy, which is particularly frequent in older patients with diabetes [25, 26], was both associated with negative diabetes-specific events such as hypoglycemia and negative health events like falls, hospitalization, and mortality. Huang et al. reported that regimens with at least four prescribed medications were significantly associated with an increased risk of falls [27]. Concerning urinary incontinence, a review and meta-analysis showed that it affected more than one-third of women aged 55 to 106 years old [28]. Age and diabetes were two of the main factors influencing urinary incontinence. Moreover, these geriatric syndromes occurred at an earlier age compared to patients without diabetes, as found in a previous study [29].

Other factors were not significantly different in patients with diabetes compared to patients without diabetes in our study, although these factors are considered to be related to both diabetes and falls. These include orthostatic hypotension [30, 31], hearing and visual impairments, and depression. Underdiagnosis of depression in older adults and self-reporting of visual impairment and hearing loss may partly explain these results. However, poorer performance in the SLS test and other parameters like gait and balance disorders, use of walking aid, and inability to leave home alone was more frequent for patients with diabetes, although they were younger compared to patients without diabetes.

Our study suggests that management of geriatric syndromes in older patients with diabetes should be considered at the same time and with the same care as potential microvascular and macrovascular complications of diabetes mellitus [25, 32].

This study is a large real-life database to assess risk factors for falls in older patients who have already fallen and to clarify the impact of diabetes on this specific population. It involves over 1500 patients, 300 of whom had diabetes.

Our study has several limitations. Some data such as the number of previous falls or the presence of neurosensory disorders are evaluated by self-assessment. This can lead to recall bias. Despite the interesting size of our population, this study remains a single-center study and reflects the population treated in a tertiary hospital. It would be interesting to validate these results in a multicenter cohort.

Care plan for older patients with diabetes should be defined in accordance with the patient's geriatric profile depending on whether they are robust, frail, or dependent. In the case of frailty or dependency, a joint evaluation between the diabetologist and the geriatrician is useful for improving management and anticipation of the risk of falls and other geriatric syndromes [33, 34]. Further studies are needed to assess the impact of a geriatric assessment to establish a personalized care plan on quality of life, risk of falls, and risk of hospitalization in older patients with diabetes.

## 5. Conclusion

Patients with diabetes represent nearly 20% of the older falling population. Several complications of diabetes as well as geriatric syndromes are risk factors for falls. Older patients with diabetes had more geriatric syndromes and more comorbidities, which make them more at risk of adverse events, including falls, compared to patients without diabetes while they are younger. In the context of an aging worldwide population, special attention should be paid to the geriatric assessment of older patients with diabetes to avoid negative health events such as falls and to personalize the patients' therapeutic goals according to their aging trajectories.

## Figures and Tables

**Table 1:** Demographical, social, and clinical characteristics.

	**Total (** **n** = 1520**)**	**Patients with diabetes (** **n** = 304**)**	**Patients without diabetes (** **n** = 1216**)**	**p** ** value**
Demographical and social characteristics				
Age, years	81.4 ± 6.4	79.4 ± 6.1	81.9 ± 6.4	< 0.0001
Sex, female, *N* (%)	1097 (72.2)	212 (70)	885 (73)	0.29
High level of education, *N* (%)	535/1319 (40.6)	76/265 (28.7)	459/1054 (43.6)	< 0.0001
Living alone, *N* (%)	794/1516 (52.4)	148/304 (48.7)	646/1212 (53.3)	0.15
Living in an institution, *N* (%)	200/1515 (13.2)	29/303 (9.6)	171/1212 (14.1)	0.037
Alcohol consumption, *N* (%)	276/1505 (18.3)	53/299 (17.7)	223/1206 (18.5)	0.76
Current tobacco consumption, *N* (%)	150/1508 (9.9)	33/300 (11)	117/1208 (9.7)	0.50
Clinical characteristics and comorbidities				
BMI, kg/m^2^	26.4 ± 5.2	28.7 ± 5.5	25.8 ± 4.9	< 0.0001
Diabetes, *N* (%)	304 (20)	—	—	
Hypertension, *N* (%)	990 (65.1)	239/304 (78.6)	751/1216 (61.8)	< 0.0001
Orthostatic hypotension, *N* (%)	439/1394 (31.5)	88/269 (32.7)	351/1125 (31.2)	0.63
Heart disease, *N* (%)	445/1516 (29.4)	109/304 (35.9)	336/1212 (27.7)	0.005
Arrhythmias, *N* (%)	372/1518 (24.5)	80/304 (26.3)	292/1214 (24)	0.41
History of stroke, *N* (%)	257/1519 (16.9)	65/303 (21.5)	192/1216 (15.8)	0.019
Chronic respiratory disease, *N* (%)	180/1514 (11.9)	48/303 (15.8)	132/1211 (10.9)	0.017
Parkinsonian syndrome, *N* (%)	225/1509 (14.9)	52/302 (17.2)	173/1207 (14.3)	0.21
Neuropathy, *N* (%)	158/1395 (11.3)	70/282 (24.8)	87/1113 (7.8)	< 0.0001
Foot disorders, *N* (%)	861/1332 (64.6)	164/279 (58.7)	698/1053 (66.3)	0.053
Osteoarthritis, *N* (%)	792/1516 (52.2)	150/302 (49.7)	642/1214 (52.9)	0.32
Osteoporosis, *N* (%)	294/1473 (19.9)	37/293 (12.6)	257/1180 (21.8)	< 0.0001
History of fractures, *N* (%)	770/1507 (51.1)	148/302 (39)	622/1205 (51.6)	0.42

*Note:* Values are expressed as number (%) or mean ± standard deviation.

Abbreviation: BMI, body mass index.

**Table 2:** Falls and other geriatric syndromes in the population study.

	**Total (** **n** = 1520**)**	**Patients with diabetes (** **n** = 304**)**	**Patients without diabetes (** **n** = 1216**)**	**p** ** value**
Geriatric syndromes				
ADL score, points	5.2 ± 1.2	4.8 ± 1.3	5.2 ± 1.1	< 0.0001
Hearing impairment, *N* (%)	730/1478 (49.4)	148/295 (50.2)	582/1183 (49.2)	0.765
Visual impairment, *N* (%)	823/1481 (55.6)	171/295 (58)	652/1186 (55)	0.355
MMSE score, points	24.8 ± 4.9	23.8 ± 5	25 ± 4.8	< 0.0001
Cognitive decline, *N* (%)	542/1512 (35.8)	138/303 (45.5)	404/1209 (33.4)	< 0.0001
Depression, *N* (%)	455/1514 (30.1)	81/302 (26.8)	374/1212 (30.9)	0.171
Number of daily medications, *N*	7.7 ± 3.4	9.6 ± 3.4	7.2 ± 3.3	< 0.0001
Polypharmacy (≥ 5 medications), *N* (%)	1259/1518 (83)	285/304 (93.8)	974/1214 (80.2)	< 0.0001
High polypharmacy (≥ 9 medications), *N* (%)	583/1518 (38.4)	186/304 (61.2)	397/1214 (33)	< 0.0001
At least one psychotropic drug, *N* (%)	832/1518 (54.2)	169/304 (55.6)	663/1214 (54.6)	0.76
At least one environmental risk factor, *N* (%)	1032/1350 (76.4)	201/270 (74.4)	831/1080 (77)	0.39
Urinary disorders, *N* (%)	414/1507 (27.5)	99/303 (32.7)	315/1204 (26.2)	0.023
Falls and associated factors				
Number of falls in the last 6 months, *N* (%)	3 ± 3.2	3.2 ± 3.2	2.9 ± 3.2	0.01
None	297/1520 (19.5)	41/304 (13.5)	256/1216 (21)	0.004
1	328/1520 (21.6)	61/304 (20.1)	267/1216 (22)	
2 or more	882/1520 (58)	199/304 (65.5)	683/1216 (56.2)	
Proprioceptive disorders, *N* (%)	805/1381 (58.3)	203/278 (73)	602/1103 (54.6)	< 0.0001
Balance disorders, *N* (%)	1059/1486 (71.3)	230/297 (77.4)	829/1189 (69.7)	0.009
Gait disorders, *N* (%)	1242/1494 (83.1)	264/300 (88)	978/1194 (82)	0.012
Timed up and go test > 20 s, *N* (%)	609/1022 (59.6)	132/205 (64.4)	477/817 (58.4)	0.117
Single-leg stance test > 5 s, *N* (%)	230/1376 (16.7)	33/277 (11.7)	197/1099 (17.9)	0.017
Fear of falling, *N* (%)	1149/1496 (76.8)	241/298 (80.9)	908/1198 (75.8)	0.063
Ability to go out alone, *N* (%)	704/1470 (47.9)	119/294 (40.5)	585/1176 (49.7)	0.004
Lying on the floor ≥ 1 h, *N* (%)	302/1364 (22.1)	71/270 (26.3)	231/1094 (21.1)	0.066
Need for walking aids, *N* (%)	693/1494 (46.4)	154/299 (51.5)	539/1195 (45.1)	0.047

*Note:* Values are expressed as numbers (%) or mean ± standard deviation.

Abbreviations: ADL, activities of daily living; MMSE, Mini-Mental State Examination.

## Data Availability

The data that support the findings of our study are available from the corresponding author upon reasonable request.

## Nomenclature

ADLs: activities of daily living
BMI: body mass index
CERIM: Center for Research and Studies in Medical Informatics
CI: confidence interval
GRECO: Groupe de Réflexion sur les Evaluations Cognitives
PM&R: physical medicine and rehabilitation
MMSE: Mini-Mental State Examination
RR: risk ratio
SLS: single-leg stance
TUG: timed up and go

## Supporting Information

Supporting Information Additional supporting information can be found online in the Supporting Information section. Graphical abstract legend: Although younger, patients with diabetes had more fall risk factors and geriatric syndromes which make them more at risk of adverse events including falls compared to patients without diabetes.
