# Supplementary material for: Fall Risk Factors and Other Geriatric Syndromes in Older Adults With Diabetes: Experience of a Multidisciplinary Fall Consultation
Source: J Diabetes Res. 2025 Aug 21;2025:6145818. doi: 10.1155/jdr/6145818 (PMC12393927; doi:10.1155/jdr/6145818)
Supplement: Supporting Information — Additional supporting information can be found online in the Supporting Information section. Graphical abstract legend: Although younger, patients with diabetes had more fall risk factors and geriatric syndromes which make them more at risk of adverse events including falls compared to patients without diabetes. [file 6145818.f1.pdf]

Patients with diabetes experience accelerated aging trajectories

- Retrospective, cohort study at Lille University Hospital Geriatrics Department, France
- Inclusion criteria:
  - any patients aged 65 and over
  - consulting for multidisciplinary fall risk assessment
  - between 2005 and 2015.
- 1520 patients included → 20% had diabetes
- Patients with diabetes were younger than patients without diabetes (mean age respectively: 79.4+/-6.1 years versus 81.9+/-6.4 years, p<0.001).

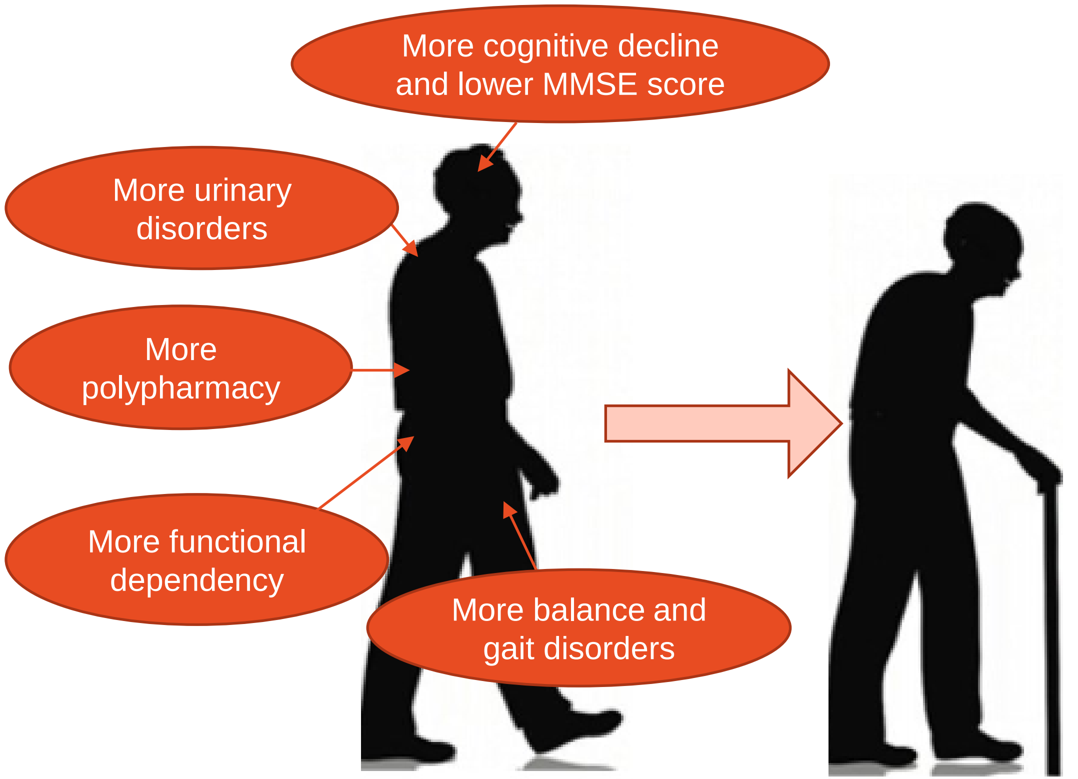

Although younger, patients with diabetes had more fall risk factors and geriatric syndromes which make them more at risk of adverse events including falls compared to patients without diabetes.
